# Carboxypeptidase vitellogenic like facilitates resistance to CDK4/6 inhibitors in breast cancer

**DOI:** 10.1111/1759-7714.14829

**Published:** 2023-02-24

**Authors:** Xiang Zhu, Xiaojie Xu, Lin Zhang, Xuhui Yang

**Affiliations:** ^1^ Department of Cellular Engineering Lab Beijing Institute of Biotechnology Beijing China; ^2^ Department of Outpatient Service 986th Hospital Affiliated to Air Force Medical University Xi'an China; ^3^ Department of Oncology, The Fifth Medical Center Chinese PLA General Hospital and Chinese PLA Medical School Beijing China

**Keywords:** breast cancer, CDK4/6 inhibitor, CPVL, resistance

## Abstract

**Objective:**

Inhibitors of cyclin‐dependent kinase 4 and 6 (CDK4/6) are targeted therapeutic drugs for breast cancer treatment. The mechanism of resistance to these inhibitors requires further investigation.

**Methods:**

We used bioinformatics to screen differentially expressed genes between cells that were susceptible and resistant to CDK4/6 inhibitors. Quantitative real‐time PCR (qRT‐PCR) was used to identify gene expressions in different cell lines. Cell viability, colony formation, cell cycle, and apoptosis assays were used to evaluate the effect of carboxypeptidase vitellogenic like (CPVL) on breast cancer cells under the condition of CDK4/6 inhibitors. Gene set enrichment analysis (GSEA) suggested the potential regulatory pathway of CPVL in breast cancer. Xenograft formation assay was conducted in nude mice to study the role of CPVL in vivo.

**Results:**

Based on bioinformatics analysis and qRT‐PCR, CPVL was identified more abundantly in cells that were resistant than sensitive to CDK4/6 inhibitors. Overexpressed or knocked down CPVL regulated the effects of CDK4/6 inhibitors in resistant cell lines. GSEA showed that resistance might be induced by CPVL through altered phosphatase and tensin homolog (PTEN)‐related pathways. Our findings showed that CPVL negatively regulates PTEN to impact the anticancer effects of CDK4/6 inhibitors in vitro and in vivo.

**Conclusion:**

CPVL might be a key factor in regulating breast cancer resistance to CDK4/6 inhibitors.

## Graphical abstract

The mechanism diagram of this study. Carboxypeptidase vitellogenic like negatively regulates phosphatase and tensin homolog to impact the anticancer effects of cyclin‐dependent kinase 4 and 6 inhibitors in breast cancer.

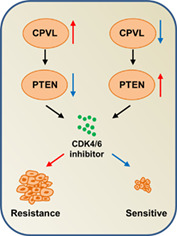

## INTRODUCTION

Breast cancer threatens the lives of women worldwide, as 600 000 die from it and ~2.1 million women are annually diagnosed for the first time. As understanding of breast cancer, has progressed, the 5‐year survival rate has reached 90%, and only 25% of cancers reach the advanced stage. Among breast cancer subtypes, 60–70% are estrogen receptor (ER)‐positive.^1^, ^2^ Cyclin‐dependent kinase (CDK) 4/6 inhibitors combined with endocrine therapy are effective against ER‐positive breast cancer.^3^, ^4^, ^5^, ^6^ However, breast cancer has become resistant to these inhibitors, leading to recurrence and catastrophic events.^7^, ^8^

Carboxypeptidase is an enzyme that splits a single amino acid from the carboxyl terminus of a protein or peptide. Carboxypeptidases activate or inactivate angiotensin, bradykinin, and other bioactive peptides. The probable serine carboxypeptidase vitellogenic like (CPVL) comprises 477 amino acids and belongs to the carboxypeptidase family.^9^, ^10^ Little is known about CPVL due to a paucity of studies.^11^, ^12^, ^13^ However, CPVL promotes glioma progression through Bruton tyrosine kinase/adenovirus early region 1A‐binding protein p300‐mediated reduction in signal transducer and activator of transcription 1 phosphorylation. CPVL is a promising marker of gastric cancer and its gene mutations are associated with type 2 diabetic retinopathy. However, the role of CPVL in breast cancer treatment or drug resistance remains unknown.

We found using datasets associated with resistance to CDK4/6 inhibitors in the Gene Expression Omnibus (GEO) that more CPVL was expressed in resistant, than sensitive breast cancer cells, implying a potential role in regulating drug resistance. More importantly, we found that CPVL promoted drug resistance to CDK4/6 inhibitors by downregulating phosphatase and tensin homolog (PTEN) in vivo and in vitro, indicating that CPVL promotes breast cancer resistance to CDK4/6 inhibitors.

## MATERIALS AND METHODS

### Reagents

The following were purchased from the sources shown: reagents and culture media (Thermo Fisher Scientific Inc.), 1:1000‐diluted anti‐β‐actin (sc‐47 778; Santa Cruz Biotechnology Inc.), 1:1000‐diluted anti‐Flag (A8592; Sigma‐Aldrich Corp.), and 1:500‐diluted anti‐CPVL (ab180147), 1:500‐diluted anti‐p‐Rb (ab184796), 1:500‐diluted anti‐PTEN (ab267787), and 1:500‐diluted anti‐cyclin E1 (ab33911; all from Abcam).

### Cell culture and establishment of resistant cell lines

MCF7 and ZR75‐1 cells were cultured at 37°C and 5% CO_2_ in Dulbecco's modified Eagle's medium (DMEM) containing 100 IU penicillin, 100 mg streptomycin, and 19% fetal bovine serum. We generated cell lines that were resistant to CDK4/6 inhibitors by exposing them to increasing concentrations of palbociclib or ribociclib until the cells clearly remained alive.

### Analysis of GEO and TCGA‐BRCA datasets

The datasets GSE98987, GSE117742, and GSE1117743 associated with CDK4/6 inhibitor resistance, and containing three DMSO‐ and palbociclib‐treated T47D cell samples, were downloaded from the GEO database. Data were also downloaded from The Cancer Genome Atlas (TCGA; https://portal.gdc.cancer.gov/repository)‐breast invasive carcinoma (BRCA) collection. Differentially expressed mRNAs were analyzed using the limma package in R (│log2FC│ > 1 and *p* < 0.05). Correlations between CPVL and potential mechanisms of tumorigenesis were determined using gene set enrichment analysis (GSEA).

### Quantitative real‐time PCR

Complete RNAs were collected from breast cancer cells using TRIzol, as described by the manufacturer. RNA was transcribed into cDNA using Moloney murine leukemia virus. The qPCR reaction was performed three times for each primer. Comparative Ct values were normalized to β‐actin mRNA levels. The forward and reverse (5′ → 3′) primer sequences were as follows:

APOE: GGCCTACAAATCGGAACTGGA and GGCGCTTCTGCAGGTCATCG; Beta‐actin: ATCACCATTGGCAATGAGCG and TTGA AGGTAGTTTCGTGGAT; CPVL: ATCCCGAATCAATTATAGGGGG and AAAGCTGATCCTCAGGTTCCG; DPYSL2: CAACTCCTTGCTGTCCTGTGG and CCACAGCAATGCGGCCTTTC; HDGFRP3: ATTGTGGGAAATAGAAAATAACCC and ‐TGCAGTCTTTGTCATCTTCATCT; KYNU: CCTGCTGGTGTTCCTACAAGT and AATGCCTTCATTGTCGCTTGCT.

### Transient transfections and lentivirus infection

Small interfering RNAs were transfected using VigoFect and Lipofectamine RNAiMAX plasmids, respectively, as described by the manufacturer. We transduced MCF7‐R cells with lentivirus vectors carrying CPVL short hairpin (sh) RNA to stably knock down CPVL. The target sequences of the CPVL shRNAs were GGCTGTTTCGCTCCCTATACAGA, CACCGTGAATAAGACTTACAACA, and ATCCATGTTTGGACTCTTTGTGG. The sequence for CPVL‐R (re‐expression) is mutated at the siRNA regions (Supporting Information Table S1).

### Cell viability

Cells (3 × 10^3^/well) were seeded into 96‐well plates and incubated at 37°C under a 5% CO_2_ atmosphere for 5 days. The culture medium was removed, then the cells were incubated with 100 μl of 90:10 DMEM:CCK‐8 for 1 h. The optical density (OD) of supernatants was measured.

### Colony formation, cell cycle, and apoptosis assays

Cells (3 × 10^3^) were seeded in 3.5‐cm plates and incubated for 10 days. The culture medium was discarded and the cells were washed twice with 2 ml of PBS. The cells were then fixed in 1 ml of 4% paraformaldehyde for 30 min, washed with PBS and stained with crystal violet. Colonies with diameters >1 mm were counted. The cells were fixed in ethanol (70%) for 1 h, then incubated with 5 μl of propidium iodide (PI) and 1% RNase A under darkness for 20 min. The cell cycle distribution was determined using flow cytometry (BD Biosciences). Apoptosis was assessed using Annexin V Apoptosis Detection kits (eBioscience), as described by the manufacturer.

### Western blot

Cells were disrupted in RIPA lysis buffer, then proteins in the lysates were resolved by SDS‐PAGE, blotted onto polyvinylidene fluoride membranes, then incubated with antibodies. Proteins of interest were detected as luminol‐based signals at 428 nM.

### Animal experiments

Eight‐week‐old female nude mice were injected with 1 × 10^7^ cells, followed 7 days later by palbociclib or saline once every 3 days. Tumors were measured every 3 days. The mice were sacrificed and harvested tissues were stored in liquid nitrogen. The Institutional Animal Care Committee of the Beijing Institute of Biotechnology approved and monitored all animal experiments.

### Statistical analysis

Data are shown as averages and standard deviations of triplicates. Differences between two, and among three or more groups were respectively analyzed using two‐tailed Student *t*‐tests and ANOVA. All data were statistically analyzed using GraphPad (GraphPad Software Inc.) and SPSS (IBM Corp.). Values with *p* < 0.05 were considered statistically significant.

## RESULTS

### Screening and validation of CPVL in breast cancer resistance to CDK4/6 inhibitors

**FIGURE 1:**
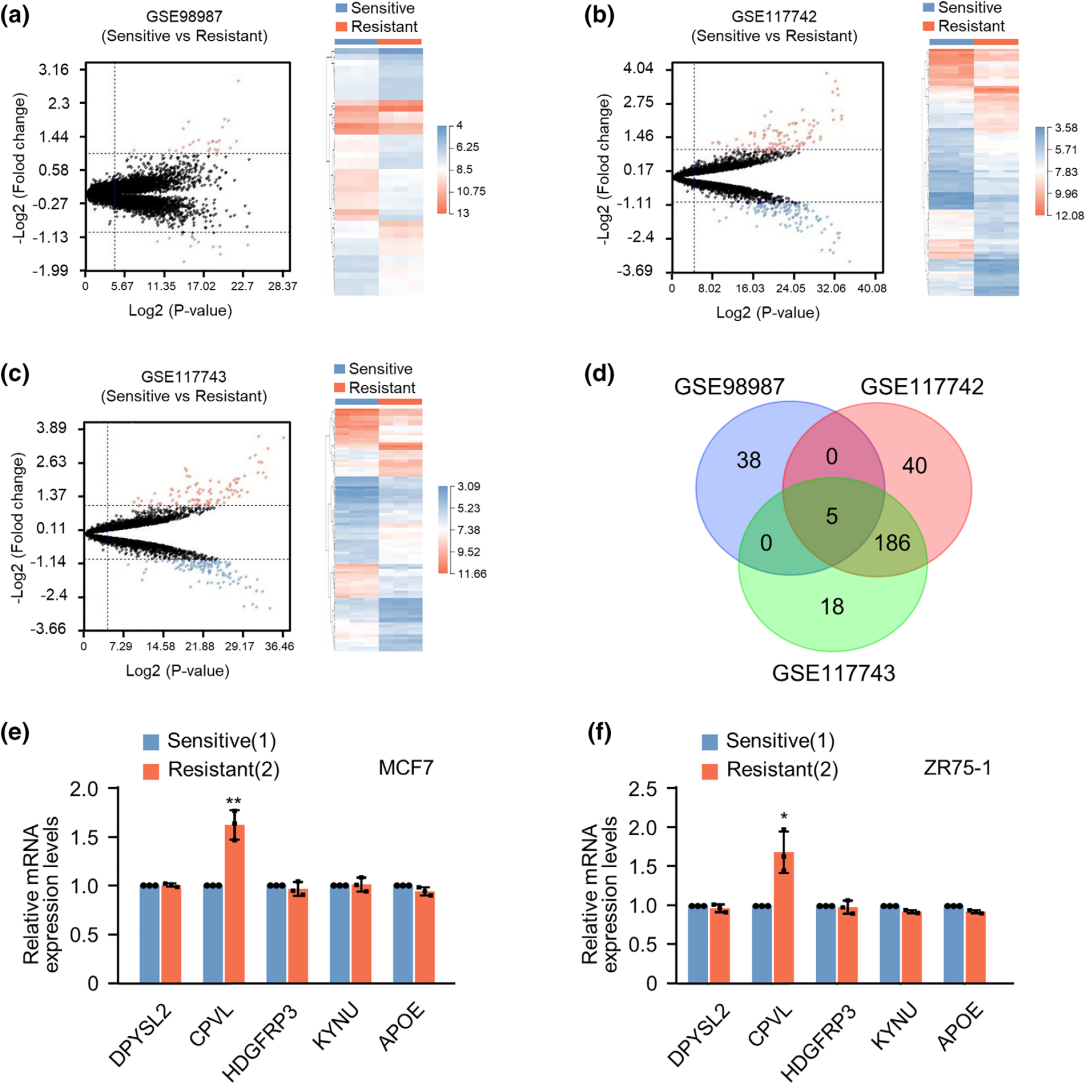
Screening and validation of CPVL in breast cancer resistant to CDK4/6 inhibitors. (a–c) Volcano plot of DEGs in GSE98987, GSE117742, and GSE117743 between in T47D cells that are sensitive and resistant to palbociclib. Values are shown as log2 of tag counts. Hierarchical clustering of RNA‐seq analysis results showed that all genes were significantly and differentially expressed. (d) Venn diagram of DEGs. Middle is intersection of the results. (e, f) Expression of DPYSL2, CPVL, HDGFRP3, KYNU, and APOE in MCF7 and ZR75‐1 sensitive and resistant cell lines to palbociclib determined by quantitative real‐time PCR. Data are shown as mean ± SD of duplicates repeated three times. All the experiments were repeated at least three times. *p < 0.05, **p < 0.01 vs. sensitive cells

We investigated factors associated with breast cancer resistance to CDK4/6 inhibitors by downloading the GSE98987, GSE117742, and GSE117743 datasets associated with breast cancer treatment by CDK4/6 inhibitors from the GEO database. We selected 28 upregulated and 15 downregulated genes from palbociclib‐treated ZR75‐1 and parent cells in GSE98987 (Figure 1a). Similar analyses revealed 102 upregulated and 129 downregulated genes in GSE117742 and 94 upregulated and 115 downregulated genes in GSE117743 (Figure 1b,c). Overlapping these results, dihydropyrimidinase‐like 2 (DPYSL2) and CPVL were upregulated, and heparin binding growth factor like 3 (HDGFL3), kynureninase (KYNU), and apolipoprotein E (APOE) were downregulated (Figure 1d). The mRNA levels of these five genes in the MCF and ZR75‐1 sensitive and resistant (MCF7‐R and ZR75‐1‐R, respectively) cells were quantified. The expression of CPVL was significantly higher in resistant, than in sensitive cell lines, whereas the mRNA levels in the other four genes did not significantly differ (Figure 1e,f). We therefore focused on the role of CPVL in breast cancer resistance to CDK4/6 inhibitors.

### CPVL regulates resistance of breast cancer cells to CDK4/6 inhibitors in vitro

**FIGURE 2:**
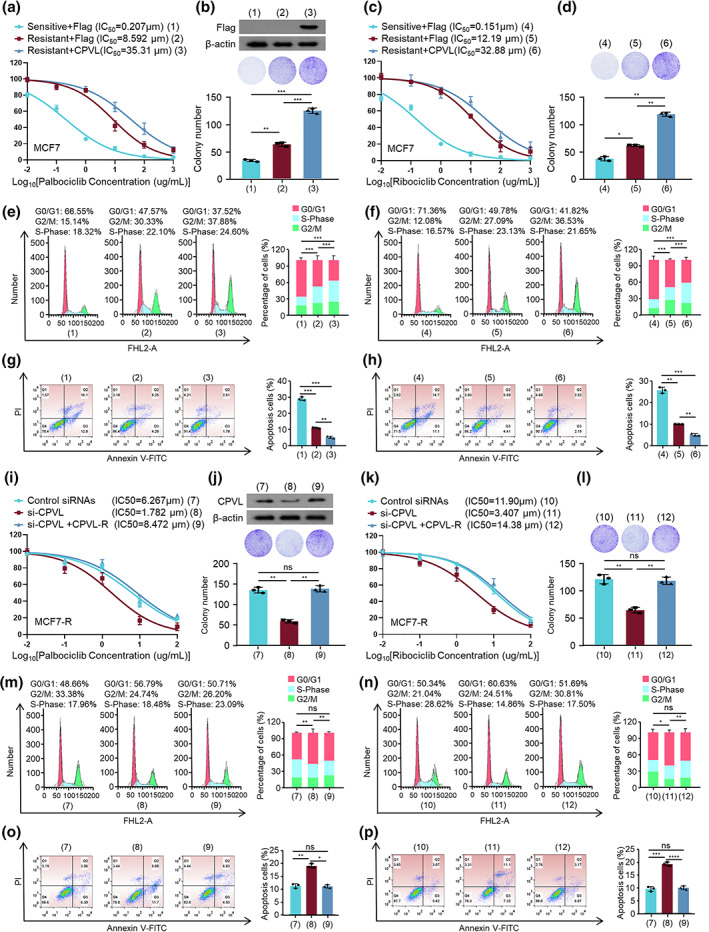
Resistance of breast cancer cells to CDK4/6 inhibitors was regulated by CPVL in vitro. Viability of MCF7‐S and MCF7‐R cells transfected with empty vector or Flag‐CPVL and incubated with gradient concentrations of palbociclib (a) or ribociclib (c). Colony formation of transfected cells incubated with 10 μM palbociclib (b) or 15 μM ribociclib (d). Cells were transfected with empty vector or Flag‐CPVL and incubated with gradient concentrations of palbociclib or ribociclib, then cell cycle distribution (e) and apoptosis (f) were evaluated by flow cytometry and ratios (%) of cells in G0/G1, S, or G2/M phases (g) and of apoptotic cells (h) were statistically analyzed. Cell viability assays of MCF7‐S and MCF7‐R cells transfected with control siRNAs or si‐CPVL or si‐CPVL plus CPVL‐R and incubated with gradient concentrations of palbociclib (i) or ribociclib (k). (j) Colony formation of cells with control siRNAs or si‐CPVL or si‐CPVL plus CPVL‐R and incubated with 10 μM palbociclib (J) or 15 μM ribociclib (l). Cells were transfected with control siRNAs or si‐CPVL or si‐CPVL plus CPVL‐R and incubated with gradient concentrations of palbociclib or ribociclib, then cell cycle distribution (m) and apoptosis (n) were evaluated by flow cytometry and ratios (%) of cells in G0/G1 or S or G2/M phases (o) and of apoptotic cells (p) were analyzed. All values are shown as mean ± SD of three sets of duplicates. *p < 0.05, **p < 0.01, ***p < 0.001, ****p < 0.0001 vs. control

We determined half‐maximal inhibitory concentrations (IC_50_) in vitro. The IC_50_ was significantly lower against palbociclib in MCF7‐sensitive than MCF7‐R cells (0.207 and 8.592 μM, respectively). The IC_50_ (35.31 μM) was also higher in cells in MCF7‐R overexpressing CPVL than in MCF7 cells expressing the empty vector (Figure 2a). We repeated the experiment using ribociclib to determine whether the effects of CPVL on CDK4/6 resistance were unique to palbociclib. However, the results were consistent with those of palbociclib (Figure 2c). The IC_50_ of palbociclib (0.1084 μM) and ribociclib (0.1838 μM) was lower in sensitive ZR75‐1 than in ZR75‐1‐R cells. The survival rates of ZR75‐1‐R cells overexpressing CPVL and exposed to palbociclib and ribociclib were better (Supporting Information Figure S1). Colony formation assays revealed better survival rates for MCF7‐R or ZR75‐1‐R than sensitive cells, and that CPVL overexpression increased colony numbers in both resistant cell lines (Figure 2b,d and Supporting Information Figure S1). The cell cycle distribution showed that both palbociclib and ribociclib blocked more sensitive, than resistant cells in the G0/G1 phase. However, overexpressed CPVL reduced the numbers of resistant cells in the G0/G1 phase (Figure 2e,f and Supporting Information Figure S1). Furthermore, overexpressed CPVL reduced the apoptosis rates of MCF7‐R and ZR75‐1‐R cells incubated with palbociclib and ribociclib (Figure 2g,h and Supporting Information Figure S1). Concordantly, overexpressed CPVL reduced apoptosis rates in MCF7‐R and ZR75‐1‐R cells incubated with palbociclib and ribociclib.

We measured the IC_50_ of palbociclib or ribociclib in MCF7‐R and ZR75‐1‐R cells transfected with CPVL siRNAs. Knockdown of CPVL reduced the resistance of MCF7‐R and ZR75‐1‐R cells to palbociclib or ribociclib, and restoring CPVL expression reversed these effects (Figure 2i,k and Supporting Information Figure S1). Colony formation, cell cycle distribution, and apoptosis rates were simultaneously detected. Knockdown of CPVL abrogated MCF7‐R or ZR75‐1‐R cell resistance to palbociclib or ribociclib (Figure 2j,l–p and Supporting Information Figure S1). In summary, CPVL promoted MCF7‐R and ZR75‐1‐R cell resistance to CDK4/6 inhibitors.

### CPVL promoted resistance to CDK4/6 inhibitors in breast cancer by reducing PTEN

**FIGURE 3:**
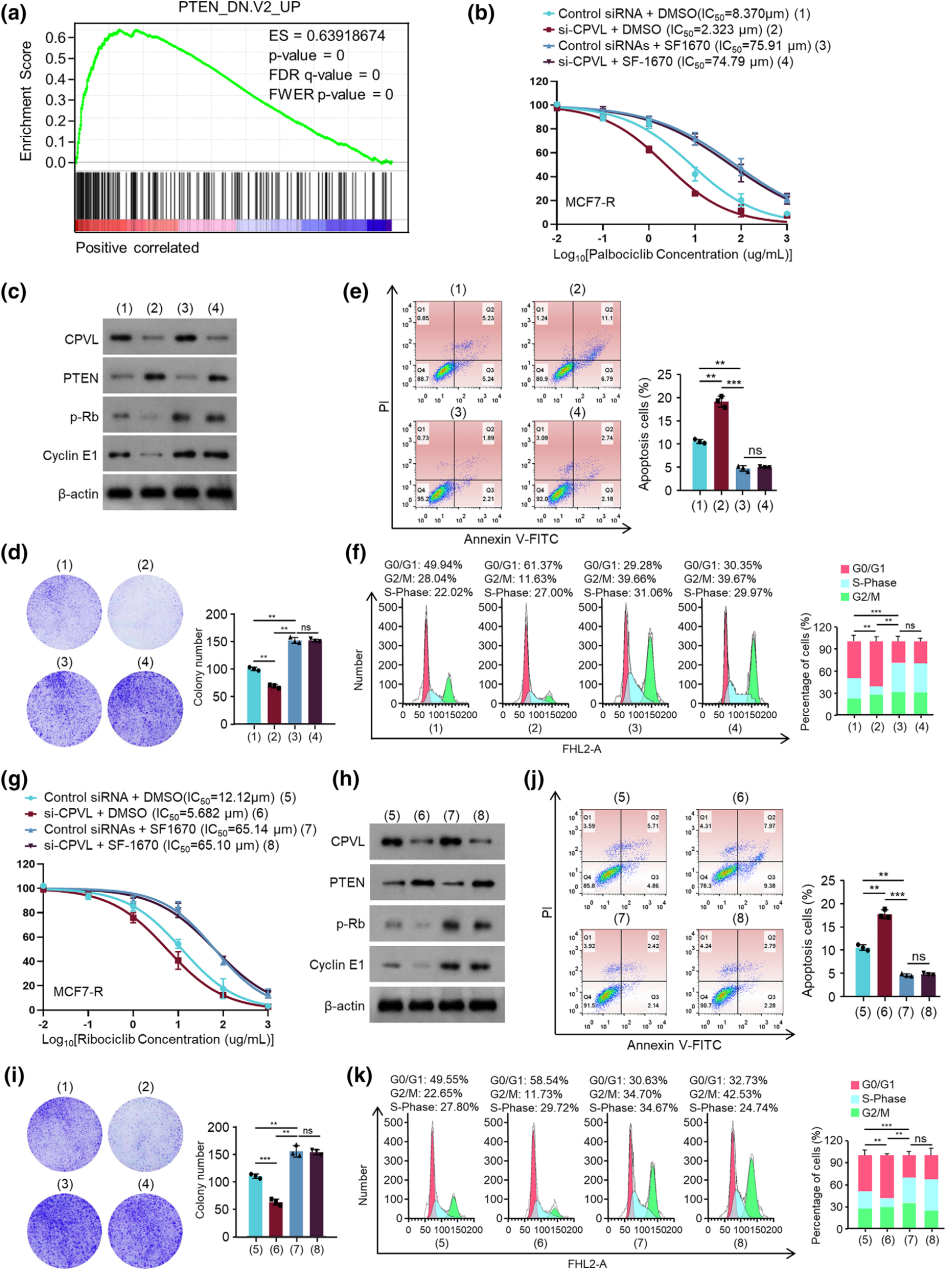
Resistance to CDK4/6 inhibitors in breast cancer is promoted by CPVL by reducing PTEN. (a) Pathways correlating with CPVL determined by GSEA. Viability of MCF7‐R cells transfected with control or si‐CPVL and incubated with DMSO or SF1760 and gradient concentrations of palbociclib (b) or ribociclib (g). Western blots of CPVL, PTEN, p‐Rb, cyclin E1, and β‐Actin protein expression in MCF7‐R cells transfected with control or si‐CPVL and incubated with 10 μM palbociclib (c) or 15 μM ribociclib (h). MCF7‐R cells were transfected with control or si‐CPVL and incubated with DMSO or SF1760 and gradient concentration of palbociclib then colony formation (d), cell cycle distribution (e), and apoptosis (f) were evaluated. MCF7‐R cells were transfected with control or si‐CPVL, then colony formation (i), cell cycle distribution (j), and apoptosis (k) were evaluated. All values are shown as mean ± SD of three repeats of duplicates. *p < 0.01, **p < 0.001 vs. control

We aimed to reveal the mechanism of CPVL in breast cancer resistance to CDK4/6 inhibitors. We investigated correlations between CPVL and potential pathways based on the TCGA‐BRCA dataset and GSEA. We found that CPVL positively correlated with the upregulated genes when PTEN was knocked down (Figure 3a). We further assessed correlations between the CPVL‐regulated resistance of MCF7‐R and ZR75‐1‐R cells to CDK4/6 inhibitors and PTEN using the PTEN‐specific activity inhibitor SF1760. The results revealed that SF1760 blocked the reduced IC_50_ caused by CPVL knockdown in MCF7‐R or ZR75‐1‐R cells exposed to palbociclib or ribociclib (Figure 3b,g and Supporting Information Figure S2). Western blotting showed that CPVL knockdown upregulated PTEN protein levels and downregulated those of p‐Rb and cyclin E1 (target genes of PTEN). SF1760 promoted p‐Rb and cyclin E1, but not PTEN. More importantly, SF1760 counteracted the effects of CPVL knockdown on the regulation of p‐Rb and cyclin E1 (Figure 3c,h and Supporting Information Figure S2). Changes in colony formation, the cell cycle distribution, and apoptotic rates caused by CPVL knockdown were reversed by adding SF1760 to MCF7‐R and ZR75‐1‐R cells incubated with palbociclib or ribociclib (Figure 3d–f,i–k and Supporting Information Figure S2), therefore SF1760 blocked CPVL and promoted resistance to CDK4/6 inhibitors by inhibiting PTEN activity in breast cancer.

### Knockdown of CPVL increases resistance to CDK4/6 inhibitors in breast cancer in vivo by enhancing PTEN

**FIGURE 4:**
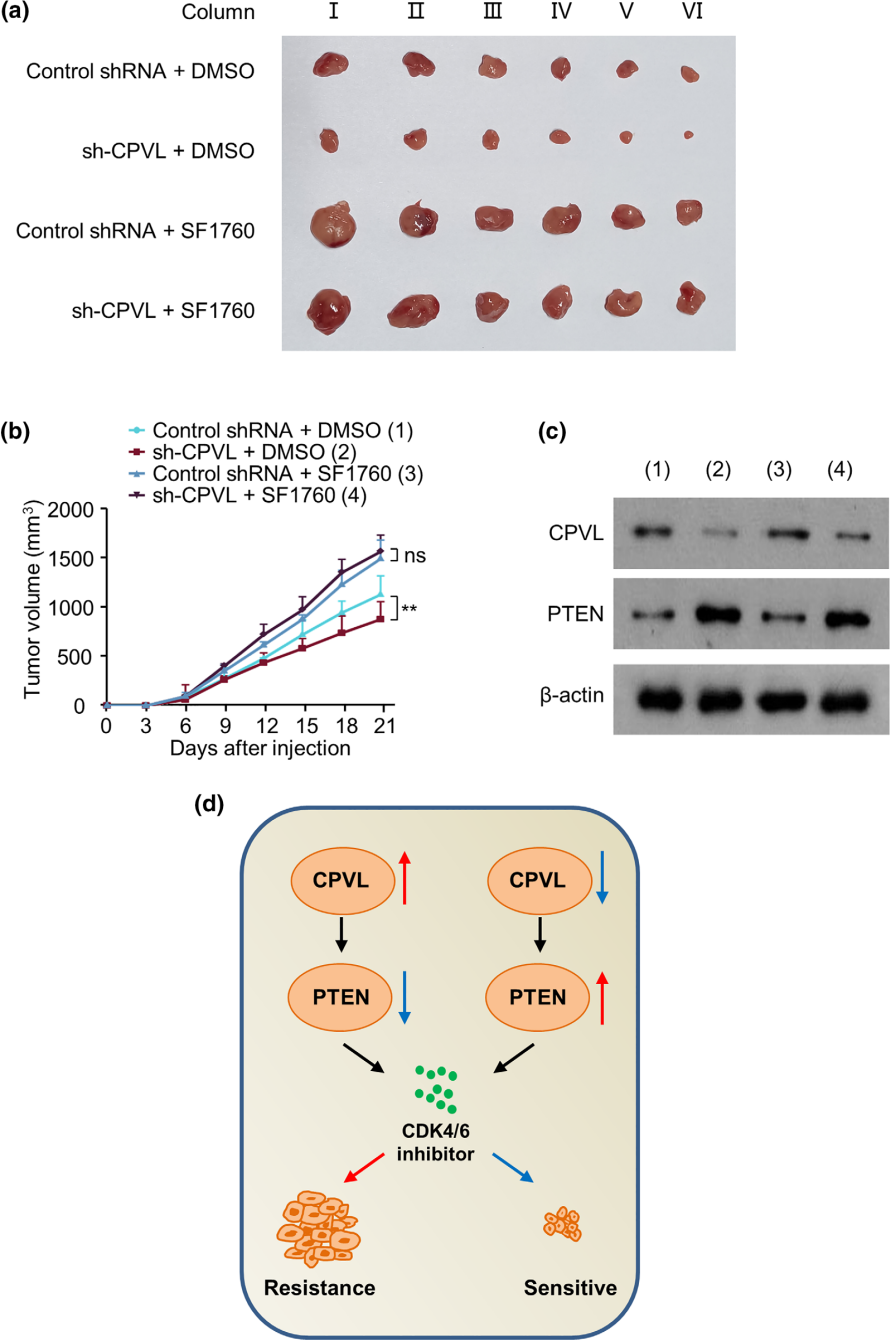
Knockdown of CPVL increases resistance to CDK4/6 inhibitors in breast cancer by enhancing PTEN in vivo. (a, b) MCF7‐R cells stably expressing control shRNA or CPVL shRNA were subcutaneously injected into the right flanks of 8‐week‐old female nude mice. Each group was injected with saline or SF1760 with palbociclib once a week after 7 days, once every 3 days. Tumor volume was measured every 3 days. (c) Western blots of representative tumor tissues to detect CPVL, PTEN, and β‐actin protein expression. (d) Schematic of regulatory network. All values are shown as mean ± SD of three repeats of duplicates. *p < 0.01 vs. control

MCF7‐R cells harboring CPVL shRNA or control shRNA were injected into the right flanks of 8‐week‐old female nude mice. Seven days later, normal saline or SF1760 was injected along with palbociclib weekly. We verified that CPVL knockdown reduced tumor volume. This weakening effect was abolished by SF1760 in vivo (Figure 4a,b). Tumors harboring CPVL shRNA expressed less CPVL and more PTEN, and adding SF1760 did not change CPVL or PTEN expression (Figure 4c). Thus, CPVL knockdown reduced breast cancer cell resistance to CDK4/6 inhibitors in vivo.

## DISCUSSION

Breast cancer has been broadly treated with CDK4/6 inhibitors for decades, and this has helped thousands of patients. The CDK4/6 inhibitors palbociclib, ribociclib, and abemaciclib are currently in use.^14^, ^15^, ^16^, ^17^, ^18^, ^19^ These inhibitors combined with endocrine therapy have been effective against breast cancer remission.^20^, ^21^ However, long‐term clinical application of these inhibitors has gradually caused the development of resistance to CDK4/6 inhibitors, regardless of congenital or long‐term drug use. In Costa's research, loss of PTEN was found in patients with breast cancer resistance to CDK4/6 inhibitors. In addition, the downregulation of p27 induced by loss of PTEN leads to the resistance of CDK4/6 inhibitors.^22^ Although the mechanisms of resistance to CDK4/6 inhibitors have been described, complex processes remain enigmatic^8^, ^14^, ^23^, ^24^, ^25^, ^26^, ^27^ therefore understanding the mechanisms of resistance to CDK4/6 inhibitors is important for prolonging the survival and improving the quality of life of patients with breast cancer.^28^ The present findings of differentially expressed genes between breast cancer cells that were resistant and sensitive to CDK4/6 inhibitors reveal that CPVL is a potential regulator of drug resistance.

The carboxypeptidase CPVL was initially discovered in human macrophages, and it is infrequently reported in tumors. Our findings of viability curves and colony formation showed that CPVL promoted breast cancer cell resistance to CDK4/6 inhibitors. Moreover, our cell cycle distribution and apoptotic findings showed that CPVL weakened the ability of CDK4/6 inhibitors to block cells in the G0/G1 phase via the negative regulation of PTEN. We also verified that CPVL knockdown regulated expressions of p‐Rb and cyclin E1, which were regulated by PTEN and implicated in cell cycle activation after G0/G1 phase. Inhibiting PTEN activity in breast cancer resulted in CPVL promoting resistance to CDK4/6 inhibitors, and CPVL knockdown decreased the tumorigenicity of CDK4/6 inhibitor‐resistant cells in mice.

In summary, we revealed the effects of CPVL on resistance to CDK4/6 inhibitors in breast cancer. CPVL reduced cell cycle arrest at the G0/G1 phase as well as apoptosis and increased resistance to CDK4/6 through PTEN. Our findings suggest a new direction for investigations into resistance, and should be informative for patients with breast cancer treated with CDK4/6 inhibitors.

## AUTHOR CONTRIBUTIONS

Xiang Zhu designed and performed the research. Lin Zhang and Xuhui Yang analyzed the data. Xiang Zhu and Xiaojie Xu wrote the manuscript. All authors read and approved the final manuscript.

## CONFLICT OF INTEREST STATEMENT

The authors declare that they have no conflict of interest.

## Supporting information


**TABLE S1.** The sequence of CPVL‐R.
**SUPPORTING INFORMATION FIGURE S1.** Carboxypeptidase vitellogenic like (CPVL) regulates resistance of ZR75‐1 cells to CDK4/6 inhibitors in vitro. Viability of ZR75‐1‐S and ZR75‐1‐R cells transfected with empty vector or Flag‐CPVL and incubated with gradient concentration of (a) palbociclib or (c) ribociclib. Colony formation of cells transfected with empty vector or Flag‐CPVL and incubated with (b) 8 μM palbociclib or (d) 11 μM ribociclib. ZR75‐1‐S and ZR75‐1‐R cells were transfected with empty vector or Flag‐CPVL and incubated with gradient concentrations of palbociclib or ribociclib then cell cycle distribution (e) and apoptosis (f) were evaluated by flow cytometry and the ratios of cells in G0/G1 or S or G2/M phases (g) and of apoptosis (h) were statistically analyzed. (i) Viability of ZR75‐1‐S and ZR75‐1‐R cells transfected with control siRNAs or si‐CPVL or si‐CPVL plus CPVL‐R and incubated with gradient concentration of palbociclib (i) or ribociclib (k). Colony formation of cells transfected with control siRNAs or si‐CPVL or si‐CPVL plus CPVL‐R and incubated with 8 μM palbociclib (j) or 11 μM ribociclib (l). Cell cycle distribution (m) and apoptosis (n) evaluated by flow cytometry and proportions of cells in G0/G1 or S or G2/M phases (o) and apoptotic cells (p) were statistically analyzed. All values are shown as mean ± SD of three sets of duplicates. *p < 0.05, **p < 0.01, ***p < 0.001, ^****^ *p* < 0.0001 vs. control

**SUPPORTING INFORMATION FIGURE S2.** Carboxypeptidase vitellogenic like (CPVL) promotes resistance to CDK4/6 inhibitors in ZR75‐1 cells by reducing PTEN. Viability of ZR75‐1‐R cells transfected with control or si‐CPVL and incubated with DMSO or SF1760 and gradient concentrations of palbociclib (a) and ribociclib (f). (b) Western blots of CPVL, PTEN, p‐Rb, cyclin E1, β‐actin protein expression in ZR75‐1‐R cells transfected with control or si‐CPVL and incubated with DMSO or SF1760 and 8 μM palbociclib (b) or 11 μM ribociclib (g). ZR75‐1‐R cells were transfected with control or si‐CPVL and incubated with DMSO or SF1760, then colony formation (c), cell cycle distribution (d), and apoptosis (e) were evaluated. (h–j) ZR75‐1‐R cells were transfected with control or si‐CPVL and incubated with DMSO or SF1760 and 8 μM palbociclib or 11 μM ribociclib then colony formation (h), cell cycle distribution (i), and apoptosis (j) were evaluated. All values are shown as mean ± SD of three sets of duplicates. *p < 0.05, **p < 0.01, ***p < 0.001, ^****^ *p* < 0.0001 vs. control
