# Supplementary figures and images for: Carboxypeptidase vitellogenic like facilitates resistance to CDK4/6 inhibitors in breast cancer
Source: Thorac Cancer. 2023 Feb 24;14(11):983–91. doi: 10.1111/1759-7714.14829 (PMC10101830; doi:10.1111/1759-7714.14829)

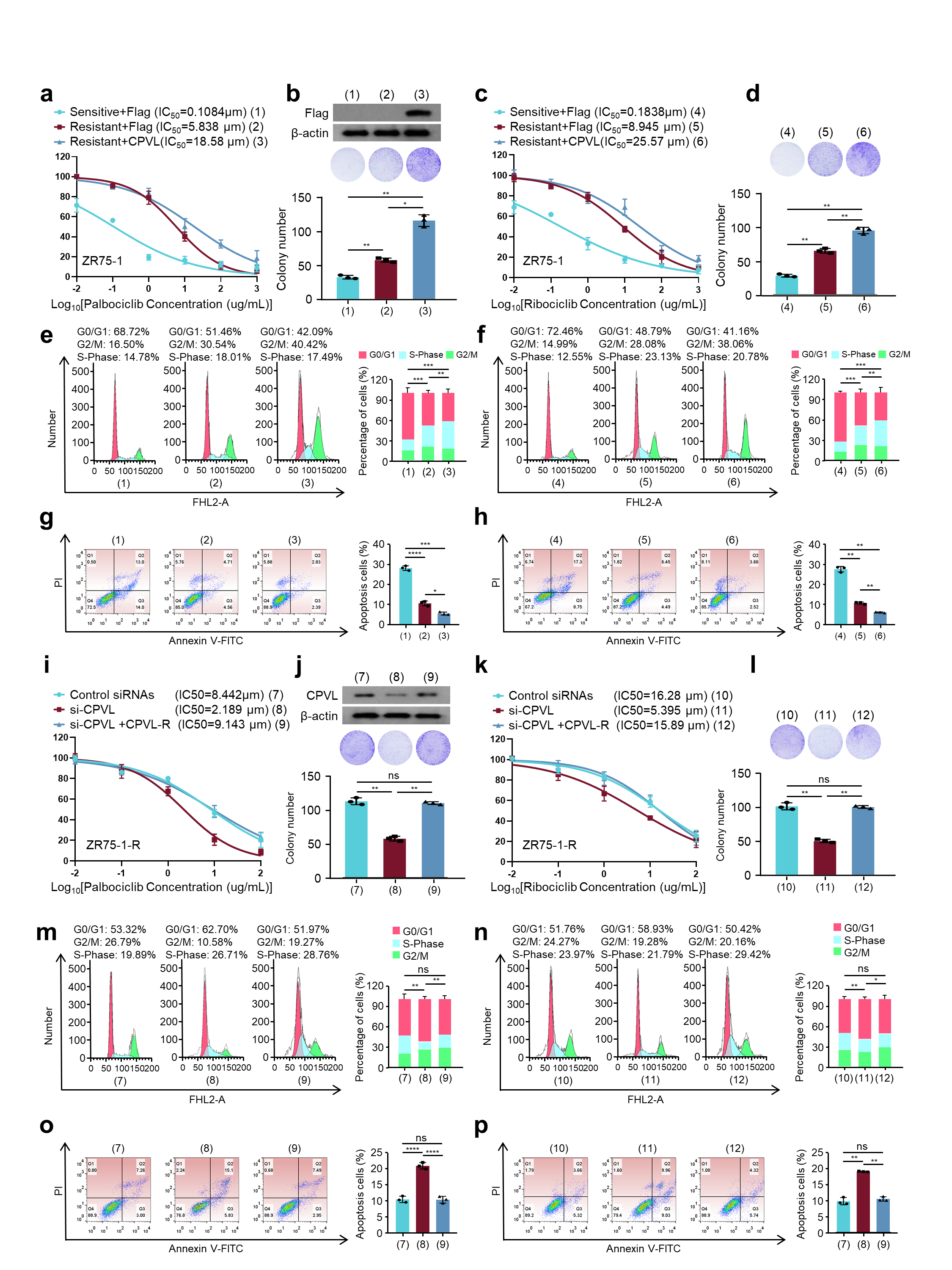

Supplement: Supplementary file 1 — TABLE S1. The sequence of CPVL‐R. SUPPORTING INFORMATION FIGURE S1. Carboxypeptidase vitellogenic like (CPVL) regulates resistance of ZR75‐1 cells to CDK4/6 inhibitors in vitro. Viability of ZR75‐1‐S and ZR75‐1‐R cells transfected with empty vector or Flag‐CPVL and incubated with gradient concentration of (a) palbociclib or (c) ribociclib. Colony formation of cells transfected with empty vector or Flag‐CPVL and incubated with (b) 8 μM palbociclib or (d) 11 μM ribociclib. ZR75‐1‐S and ZR75‐1‐R cells were transfected with empty vector or Flag‐CPVL and incubated with gradient concentrations of palbociclib or ribociclib then cell cycle distribution (e) and apoptosis (f) were evaluated by flow cytometry and the ratios of cells in G0/G1 or S or G2/M phases (g) and of apoptosis (h) were statistically analyzed. (i) Viability of ZR75‐1‐S and ZR75‐1‐R cells transfected with control siRNAs or si‐CPVL or si‐CPVL plus CPVL‐R and incubated with gradient concentration of palbociclib (i) or ribociclib (k). Colony formation of cells transfected with control siRNAs or si‐CPVL or si‐CPVL plus CPVL‐R and incubated with 8 μM palbociclib (j) or 11 μM ribociclib (l). Cell cycle distribution (m) and apoptosis (n) evaluated by flow cytometry and proportions of cells in G0/G1 or S or G2/M phases (o) and apoptotic cells (p) were statistically analyzed. All values are shown as mean ± SD of three sets of duplicates. *p < 0.05, **p < 0.01, ***p < 0.001, **** p < 0.0001 vs. control [file TCA-14-983-s002.TIF]

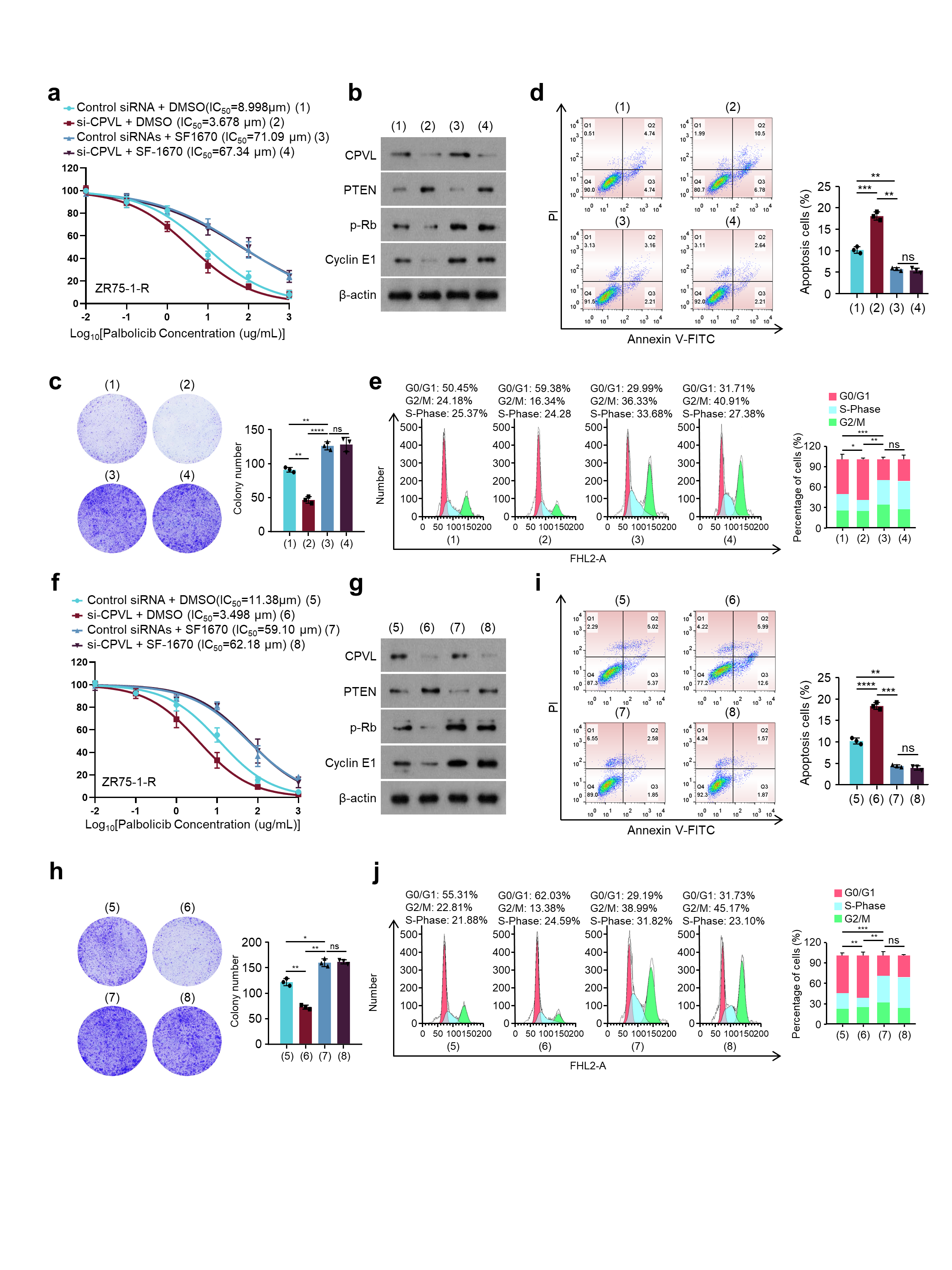

Supplement: Supplementary file 2 — SUPPORTING INFORMATION FIGURE S2. Carboxypeptidase vitellogenic like (CPVL) promotes resistance to CDK4/6 inhibitors in ZR75‐1 cells by reducing PTEN. Viability of ZR75‐1‐R cells transfected with control or si‐CPVL and incubated with DMSO or SF1760 and gradient concentrations of palbociclib (a) and ribociclib (f). (b) Western blots of CPVL, PTEN, p‐Rb, cyclin E1, β‐actin protein expression in ZR75‐1‐R cells transfected with control or si‐CPVL and incubated with DMSO or SF1760 and 8 μM palbociclib (b) or 11 μM ribociclib (g). ZR75‐1‐R cells were transfected with control or si‐CPVL and incubated with DMSO or SF1760, then colony formation (c), cell cycle distribution (d), and apoptosis (e) were evaluated. (h–j) ZR75‐1‐R cells were transfected with control or si‐CPVL and incubated with DMSO or SF1760 and 8 μM palbociclib or 11 μM ribociclib then colony formation (h), cell cycle distribution (i), and apoptosis (j) were evaluated. All values are shown as mean ± SD of three sets of duplicates. *p < 0.05, **p < 0.01, ***p < 0.001, **** p < 0.0001 vs. control [file TCA-14-983-s001.TIF]
